# Comparative transcriptome analysis reveals the molecular regulation underlying the adaptive mechanism of cherry (*Cerasus pseudocerasus* Lindl.) to shelter covering

**DOI:** 10.1186/s12870-019-2224-x

**Published:** 2020-01-17

**Authors:** Tian Tian, Guang Qiao, Zhuang Wen, Bin Deng, Zhilang Qiu, Yi Hong, Xiaopeng Wen

**Affiliations:** 1 Key Laboratory of Plant Resource Conservation and Germplasm Innovation in Mountainous Region (Ministry of Education), Institute of Agro-bioengineering/ College of Life Science, Guizhou University, Guiyang, 550025 People’s Republic of China; 2 Institute for Forest Resources & Environment of Guizhou/ College of Forestry, Guizhou University, Guiyang, 550025 People’s Republic of China

**Keywords:** Cherry, Rain shelter, Transcriptional regulation, Photosynthesis, Anthocyanin biosynthesis

## Abstract

**Background:**

Rain-shelter covering is widely applied during cherry fruit development in subtropical monsoon climates with the aim of decreasing the dropping and cracking of fruit caused by excessive rainfall. Under rain-shelter covering, the characteristics of the leaves and fruit of the cherry plant may adapt to the changes in the microclimate. However, the molecular mechanism underlying such adaptation remains unclear, although clarifying it may be helpful for improving the yield and quality of cherry under rain-shelter covering.

**Results:**

To better understand the regulation and adaptive mechanism of cherry under rain-shelter covering, 38,621 and 3584 differentially expressed genes were identified with a combination of Illumina HiSeq and single-molecule real-time sequencing in leaves and fruits, respectively, at three developmental stages. Among these, key genes, such as those encoding photosynthetic-antenna proteins (*Lhca* and *Lhcb*) and photosynthetic electron transporters *(PsbP*, *PsbR*, *PsbY*, and *PetF*), were up-regulated following the application of rain-shelter covering, leading to increased efficiency of light utilization. The mRNA levels of genes involved in carbon fixation, namely, *rbcL* and *rbcS*, were clearly increased compared with those under shelter-free conditions, resulting in improved CO_2_ utilization. Furthermore, the transcription levels of genes involved in chlorophyll (*hemA*, *hemN*, and *chlH*) and carotenoid synthesis (*crtB*, *PDS*, *crtISO*, and *lcyB*) in the sheltered leaves peaked earlier than those in the unsheltered leaves, thereby promoting organic matter accumulation in leaves. Remarkably, the expression levels of key genes involved in the metabolic pathways of phenylpropanoid (*PAL*, *C4H*, and *4CL*) and flavonoid (*CHS*, *CHI*, *F3’H*, *DFR*, and *ANS*) in the sheltered fruits were also up-regulated earlier than of those in the unsheltered fruits, conducive to an increase in anthocyanin content in the fruits.

**Conclusions:**

According to the physiological indicators and transcriptional expression levels of the related genes, the adaptive regulation mechanism of cherry plants was systematically revealed. These findings can help understand the effect of rain-shelter covering on Chinese cherry cultivation in rainy regions.

## Background

Cherry is a popular spring–summer fruit because of its early maturity and delicious taste, along with its antioxidant properties [1]. Cherry trees prefer environments that are sunny and without stagnant water [2]. During fruit development, they are vulnerable to the weather, such as rain and hail, which are the main causes of the severe dropping and cracking of fruit [3].

Colorless rain-shelter coverings for fruit crops such as grapes [4] and apples [5] are increasingly being used worldwide, and this may significantly enhance fruit yield [6], while having no obvious adverse effect on fruit quality [7]. However, rain-shelter covering may unavoidably alter the microclimate of the canopy, leading to a reduction in the photosynthetically active radiation (PAR) [8], which is regarded as a key constraint for vegetative growth and fruit development [9]. Therefore, flexible adjustment between light energy utilization and photoprotection is critical for plant performance and field adaptability under changing light conditions [10].

Recently, the adaptability of plants to weak light and shade conditions was documented chiefly in terms of physiological adjustment in many species, such as rubber tree [11], cotton [12], and sweet cherry [13]. It is generally accepted that the shift in the physiological adaptability of plants to adverse conditions can ultimately be attributed to alterations at the molecular level. To date, the adaptive regulatory mechanisms of plants under variable light and/or temperature conditions have been analyzed using transcriptomic strategies in many crops; for example, differentially expressed genes (DEGs) in maize were mainly involved in most assimilation processes (photosynthesis and carbon fixation pathways), namely, photosynthetic light capture via the modification of chlorophyll (Chl) biosynthesis, suggesting the complex regulatory mechanisms and interactions between cold and light signaling processes [14]. Under low-light conditions, the expression of photosystem I and II complex and electron transport-related genes increased in low-light-tolerant rice varieties; thus, the accelerated expression of photosynthesis-related genes under low light conditions contributed to the maintenance of rice yield [15]. In addition, under low light conditions, leaves need to maintain effectively antioxidant capacity to maintain their carbohydrate production level [16], which was enough to prove that adjustment or adaptation of leaves to low light conditions (hours or days) was achieved by integrating multiple signals, as shown by a study conducted on *Arabidopsis thaliana* [10]. Although the leaf adaptation to short-term light change had been considerably studied, the physiological and molecular responses of leaves to long-term weak light had not yet been unraveled so far.

Anthocyanin biosynthesis and accumulation in the fruit peel were regulated by the light intensity [17]. Owing to the frequent rain in spring, anthocyanin biosynthesis was severely inhibited by light loss of 8–22% [18], resulting in the reduction in the anthocyanin content. The flavonoid and phenylpropanoid pathways played key roles in pigment synthesis in strawberry fruit [19]. The structural genes for anthocyanin biosynthesis, such as *PAL*, *CHS*, *CHI*, *F3H*, *DFR*, *ANS*, and *UFGT*, had been reported in sweet cherry [20]. Under shelter covering, previous studies have only focused on the interaction between fruit anthocyanin contents and the transcription of related genes in grape [21]. However, to date, however, the molecular regulation of sheltered covering on the gene expression profiles of flavonoid and/or phenylpropanoid pathway has not yet been elucidated, which may be helpful for better understanding the anthocyanin accumulation.

Chinese cherry (*Cerasus pseudocerasus* Lindl.) is widely grown in Southwest China, which is characterized as a region with a subtropical monsoon, with frequent rain and hail in spring, severely constraining the expansion of the cherry industry. Previously, the use of shelter covering was substantially justified to contribute to the total Pn accumulation and fruit yield elevation, and to cause no obvious adverse effects on the vegetative growth and fruit quality of cherry [22]. To fully understand the adaptability and regulatory mechanism of cherry on microclimatic changes under shelter covering, the objectives of present study were as the following: 1) to discover the DEGs upon shelter covering via transcriptomic analysis; 2) to identify the key genes involved in growth and development; and 3) to reveal the regulatory network involved in light-harvesting, photosynthetic electronic transport, and the antioxidant system etc., which can strongly facilitate the innovative improvement of plantation systems of this fruit crop.

## Results

### Overview of the single-molecule real-time (SMRT) sequencing

**Fig. 1:**
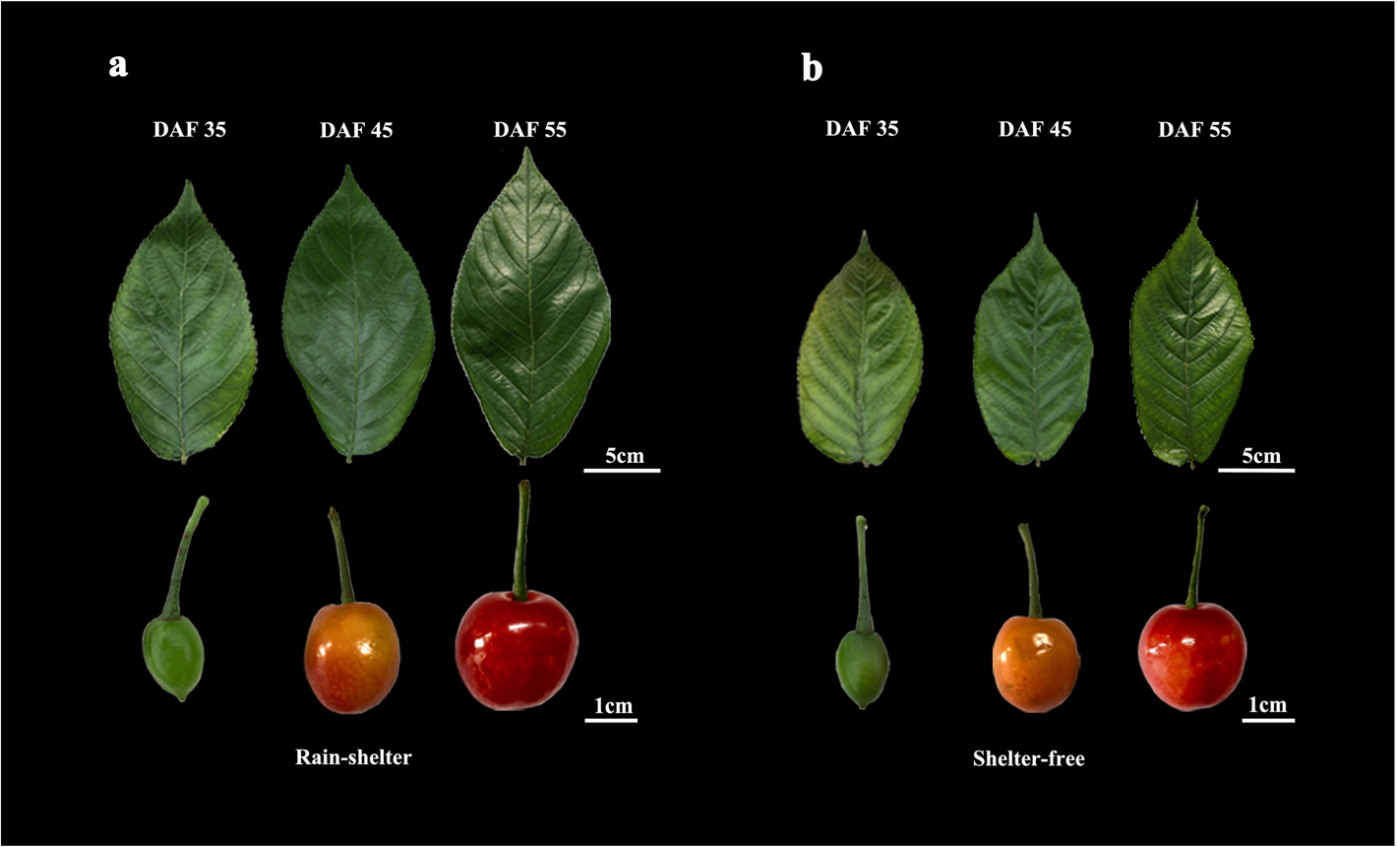
Leaves and fruits cultivated in the sheltered and unsheltered covering. The leaves and fruits of (**a**) the sheltered and (**b**) unsheltered covering in three development stages (DAF35, 45, 55) were collected for transcriptome sequencing

To obtain the gene expression profiles under sheltered and unsheltered conditions, SMRT sequencing and Illumina RNA-Seq were conducted for leaves (Fig. 1a) and fruits (Fig. 1b) on the 35th, 45th, and 55th days after flowering (DAF35, DAF45, and DAF55). In total 1,048,866 post-filter polymerase reads (21.55G) were scanned. The subreads from the same polymerase read sequence generated a circular consistent sequence (CCS), which yielded 685,339 CCS sequences. Among them, 542,795 full-length non-chimera (FLNC) sequences with 5′-primer, 3′-primer, and poly-A were obtained, including 79.20% of all CCSs being FLNCs (300–22,293 bp). This proportion varied slightly between the two tissues, at 34.96% in the leaf data set and 44.25% in the fruit data set. The mean length of the FLNC reads is presented in Additional file 2: Table S1, and the length distribution of the FLNC reads is shown in Additional file 1: Figure S1. The FLNC length of leaf and fruit libraries of > 2 kb accounted for 63.95 and 65.21% of the corresponding FLNC, respectively. Comparison between the protein-coding gene transcripts and those of FLNC revealed a strong concordance, which exhibited a better recovery of large transcripts than previous Illumina RNA-Seq data for gene model prediction, particularly in the size range of 2500–4000-bp size range [23].

### Annotation and functional classification of unigenes

**Fig. 2:**
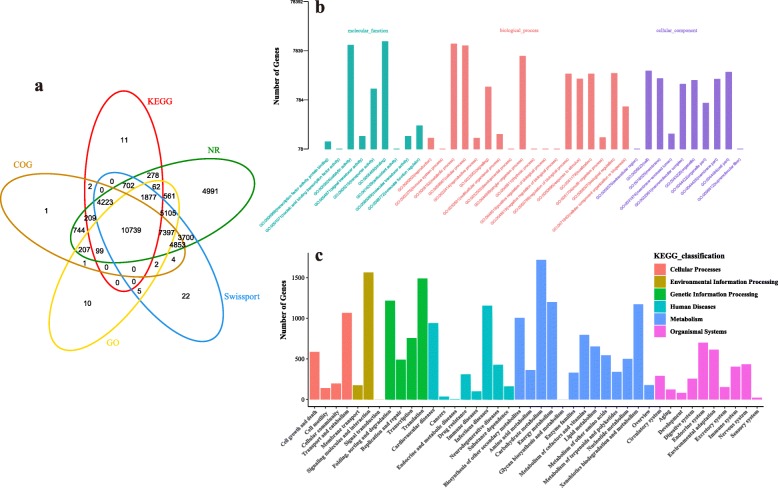
Annotation and functional classification of unigenes. **a** Venn diagram of unigenes functionally annotated for leaves and fruits. **b** Annotation of the GO database. The x-axis is the GO classification of gene function and the y-axis is the number of genes in the annotation. **c** Classification of KEGG annotation. The x-axis is the classification of gene function and the y-axis is the number of genes in the interpretation

The functions of unigenes were annotated by BLAST comparison and were predicted by comparative analysis with five databases [NCBI Nonredundant Protein Sequences (NR), Clusters of Orthologous Groups of Proteins (COG), a manually annotated and reviewed protein sequence database (Swiss-Prot), Kyoto Encyclopedia of Genes and Genomics (KEGG), Gene Ontology (GO)]. Among the 45,825 unigenes distributed to each of the databases, 45,747 (97.35% of the total) for Nr, 28,481 (60.61%) for COG, 38,629 (82.20%) for SwissProt, 18,202 (38.73%) for KEGG, and 26,065 (55.46%) for GO were investigated (Fig. 2a).

Analysis of the Nr database indicated that the highest homologies of cherry were with *Prunus mume* or with *P. persica*, with 22,919 (50.09%) and 18,545 (40.53%) unigenes annotated, respectively. In total, 28,481 annotated unigenes were classified into 37 functional groups of the three GO main categories: nine groups for molecular function (MF), 18 for biological process (BP), and 10 for cellular component (CC) (Fig. 2b, Additional file 4: Table S3). The top three GO terms for the classified genes were “protein binding” (4812), “ATP binding” (3907), and “protein kinase activity” (2272) for MF; “protein phosphorylation” (2273), “oxidation-reduction process” (1833), and “signal transduction” (1255) for BP; and “membrane” (1656), “integral component of membrane” (1571), and “nucleus” (748) for CC.

In total, 18,202 unigenes were mapped into 265 KEGG database pathways. The top three KEGG pathways were “Metabolism, Carbohydrate Metabolism” (1719); “Environmental Information Processing, Folding, Sorting, and Degradation” (1563); and “Genetic Information Processing, Translation” (1491) (Fig. 2c, Additional file 5: Table S4). The maps representing the highest number of unigenes included those of carbon metabolism (map 01200), RNA transport (map 03013), biosynthesis of amino acids (map 01230), protein processing in endoplasmic reticulum (map 04141), Spliceosome (map 03040), and starch and sucrose metabolism (map 00500).

### Functional classification of DEGs under sheltered covering

**Fig. 3:**
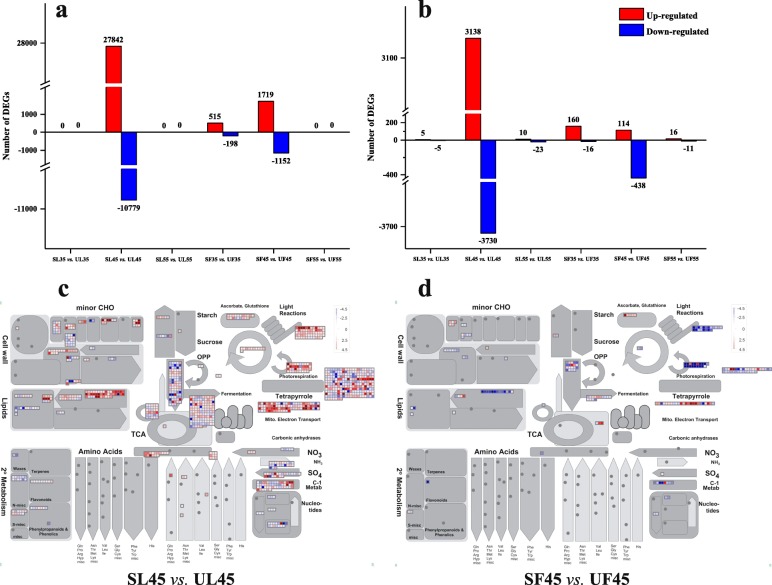
Expression patterns based on DEGs of leaves and fruits among three developmental stages. **a** DEG numbers from the GO database. **b** DEG numbers from the KEGG database. Scatter plots of GO and KEGG pathway-enrichment statistics of leaves and fruits at DAF45. **c** and **d** MapMan metabolism overview maps showing differences in transcript levels (UL45 versus SL45 and UF45 versus SF45). The Log2FPKM ratios for average transcript abundance were based on *Arabidopsis thaliana* reference genome. The resulting file was loaded into the MapMan Image Annotator module to generate the metabolism overview map. On the logarithmic color scale, blue represents down-regulated transcripts, and red represents up-regulated transcripts

After filtering the low-quality reads, 17.25 billion clean reads were acquired by Illumina RNA-Seq (Additional file 6: Table S5). According to the Illumina data, pair-wise comparisons (SL vs. UL, SF vs. UF) of gene expression among the three stages were performed. In response to microclimatic change with low-PAR conditions, in total, 38,621 (SL35 vs. UL35, SL45 vs. UL45, and SL55 vs. UL55) and 3584 (SF35 vs. UF35, SF45 vs. UF45, and SF55 vs. UF55) DEGs were detected from the GO database. Additionally, 38,621 DEGs for SL45 vs. UL45 and 2871 DEGs for SF45 vs. UF45 were acquired (Fig. 3a). Throughout the three developmental stages, a total of 6911 and 1755 DEGs were detected from the KEGG database in leaves and fruits; 6868 and 1552 DEGs for SL45 vs. UL45 and SF45 vs. UF45 were obtained, respectively (Fig. 3b). To assess the adaptation of leaves and fruits to the sheltered covering, the overall changes in biochemical pathways were analyzed using MapMan (Additional file 7: Table S6), including the DEGs of SL vs. UL and SF vs. UF at DAF45 (Fig. 3c, d). The overall changes in the biochemical pathway of these DEGs were shown by MapMan annotation. The metabolic activities of the leaves under the sheltered covering were greatly increased at DAF45, and the DEGs were assigned to different functional categories, such as photosynthesis, secondary metabolism, coenzyme metabolism, RNA biosynthesis, etc. (Fig. 3c). Under shelter covering for 45 days, the DEGs of fruit characterized by the up-regulation of secondary metabolism, cell wall organization, amino acid metabolism, etc. (Fig. 3d).

**Fig. 4:**
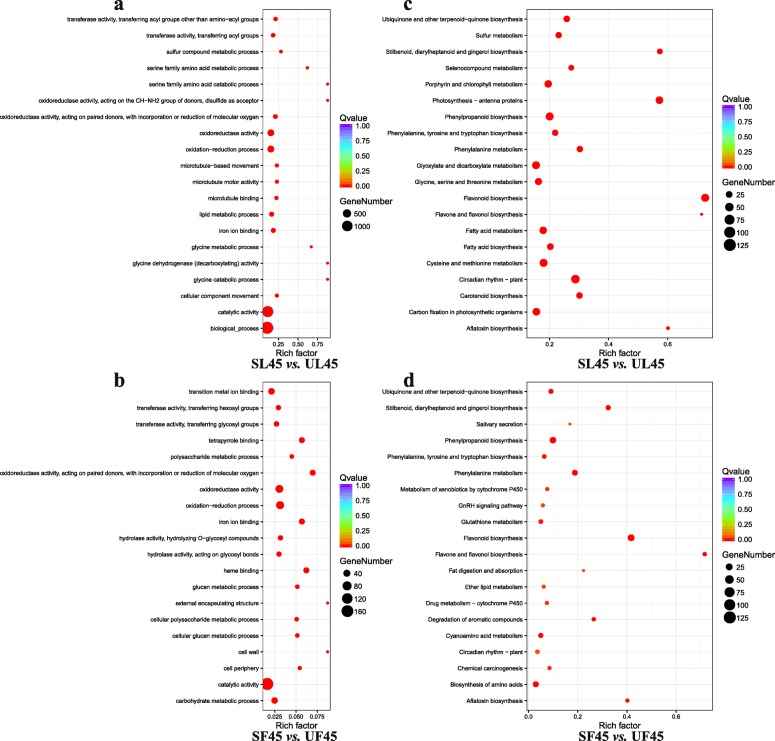
Annotation and functional classification of DEGs. **a** and **b** The top 20 up-regulated enriched GO pathways of leaves and fruits, respectively. **c** and **d** The top 20 up-regulated enriched KEGG pathways of leaves and fruits, respectively

Compared with UL and UF, the up-regulated genes from GO terms of SL were significantly enriched in “catalytic activity,” “biological process,” “oxidoreductase activity,” and “oxidation-reduction process,” among others (Fig. 4a). The up-regulated genes of SF were primarily associated with “catalytic activity,” “biological process,” “oxidoreductase activity,” and “oxidation-reduction process,” among others, at DAF45 (Fig. 4b). Up-regulated genes from KEGG terms of SL in contrast to UL included those associated with “circadian rhythm-plant,” “glyoxylate and dicarboxylate metabolism,” “porphyrin and chlorophyll metabolism,” and “carbon fixation in photosynthetic organisms,” among others (Fig. 4c); moreover, the up-regulated genes of SF were particularly associated with “biosynthesis of amino acids,” “phenylpropanoid biosynthesis,” “phenylalanine metabolism,” and “flavonoid biosynthesis,” among others, at DAF45 (Fig. 4d).

**Fig. 5:**
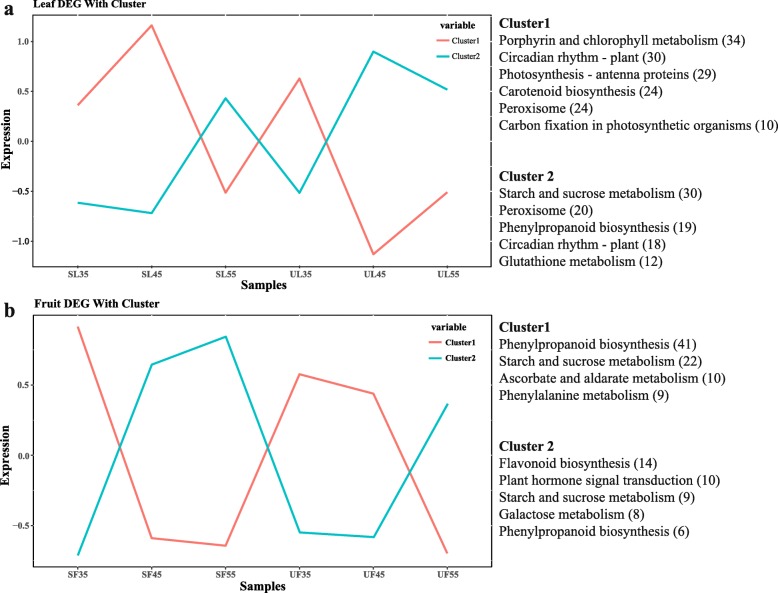
Cluster analysis of DEGs of leaves and fruits. The cluster analysis of DEGs showing significant changes in expression profiles of leaves (**a**) and fruits (**b**). Enriched KEGG pathways are listed in each cluster. Numbers in brackets indicate the quantity of DEGs enriched

Common expression patterns were employed to further analyze the DEGs between SL vs. UL and SF vs. UF at three stages; overall, 7244 (leaf) and 1707 (fruit) DEGs were placed into four clusters (Fig. 5). Most of the candidate DEGs was categorized into either leaf Cluster 1 (3796 genes) or fruit Cluster 1 (1271 genes). By KEGG pathway enrichment, the biological pathway of enrichment in each similar regulatory transcription cluster was described. The DEGs in leaf Cluster 1 exhibited peak expression at DAF45 of SL, including a broad range of genes responsible for porphyrin and cholorophyll metabolism, circadian rhythm-plant, photosynthesis-antenna proteins, carotenoid biosynthesis, peroxisome, and carbon fixation in photosynthetic organisms. The DEGs were mainly involved in photosynthesis, which reflected that leaves can maintain an effective adaptability from DAF35 to DAF45 after a long duration of rain-sheltered covering. For the top five accumulated pathways in leaf Cluster 2, the DEGs showed peak expression at DAF45 of UL, including peroxisome, phenylpropanoid biosynthesis and glutathione metabolism, and these DEGs were mainly involved in the antioxidant system (Fig. 5a). Meanwhile, for the top four enriched pathways in fruit Cluster 1, the DEGs showed peak expression at DAF35 of SF, including a broad range of genes responsible for phenylpropanoid biosynthesis, starch and sucrose metabolism, ascorbate and aldarate metabolism, and phenylalanine metabolism. Finally, for the top five enriched pathways in fruit Cluster 2, the DEGs showed peak expression at DAF55 of SF, including flavonoid biosynthesis, plant hormone signal transduction, starch and sucrose metabolism, and phenylpropanoid biosynthesis (Fig. 5b). Transcriptional results indicated that the adaptability of leaves to the microclimate was primarily attributed to the regulation of photosynthetic characteristics, assimilation, antioxidant status, and circadian rhythm. The genes implicated in the biosynthesis of sugars and anthocyanins in the sheltered fruits were up-regulated at DAF35 until DAF55, reflecting the prior accumulation of nutrition compared with that under shelter-free conditions.

**Fig. 6:**
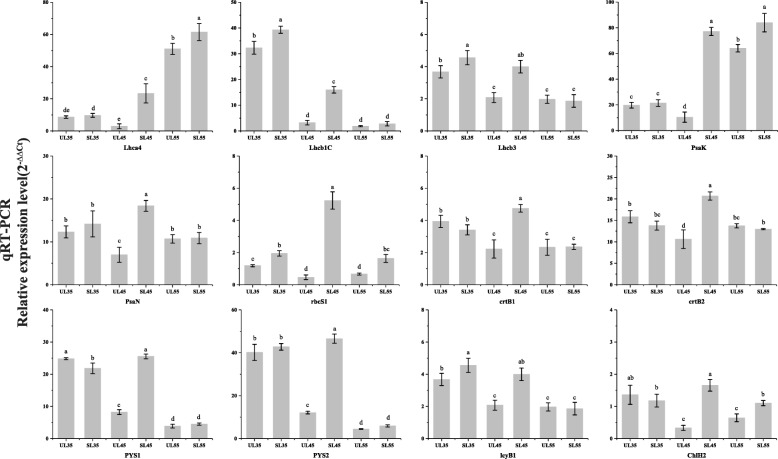
Validation of the RNA-Seq results using qRT-PCR analysis. The y-axis represents relative expression and the x-axis represents three stages of leaf development and different cultivation facilities. The standard error of the mean for three biological replicates (nested with three technical replicates) is represented by the error bars. Different letters on each symbol indicate statistically significant differences (*P* < 0.05) between two values according to ANOVA and Duncan’s new multiple range tests

To verify the authenticity of the RNA-Seq results, 12 DEGs were randomly selected and their expression profiles were analyzed by qRT-PCR. The results of qRT-PCR analysis showed that the expression profiles of these DEGs were similar to those assessed by RNA-Seq (Fig. 6), confirming the reliability and accuracy of our RNA-Seq data.

### Expression of genes involved in photosynthetic system in sheltered leaves

**Fig. 7:**
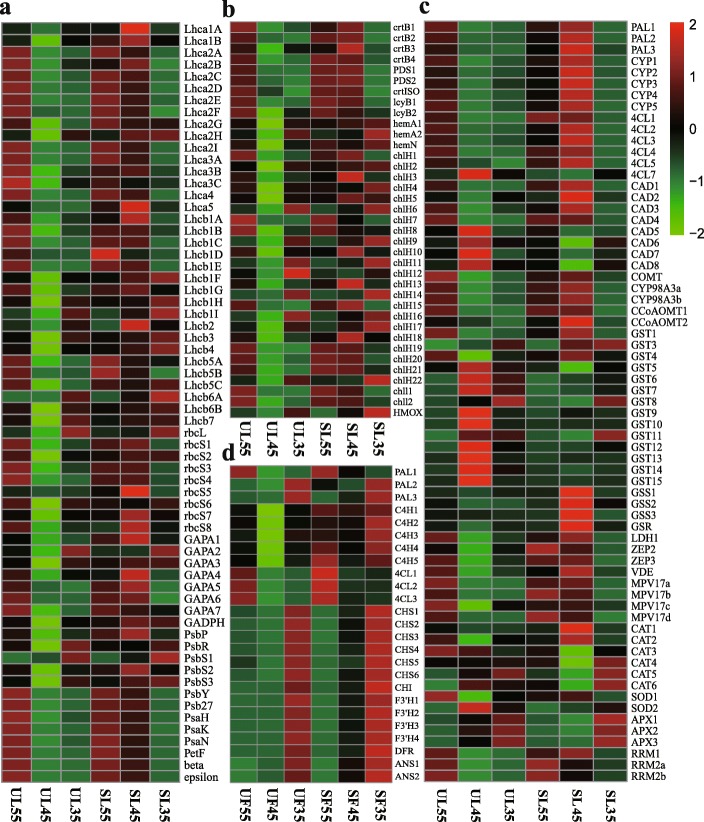
Heat maps representing the expression patterns of genes in leaves and fruits at DAF35, 45, and 55 under shelter covering and shelter-free conditions. **a** Genes involved in photosynthetic systems. **b** Genes related to chlorophyl and carotenoid synthesis. **c** Genes involved in antioxidant system. **d** Genes related to anthocyanin biosynthesis. The log2FoldChange (FPKM, Reads Per Kilobase of exon model per Million mapped reads) values were used to generate the heat maps. The x-axis indicates the sampling time, while the y-axis indicates key differentially expressed genes. The green-black-red schemes are labeled above the heat maps. Red and green represent higher and lower expression levels, respectively, than those shown in black (the black represents the value of log2 (FPKM+ 1) was zero)

**Fig. 8:**
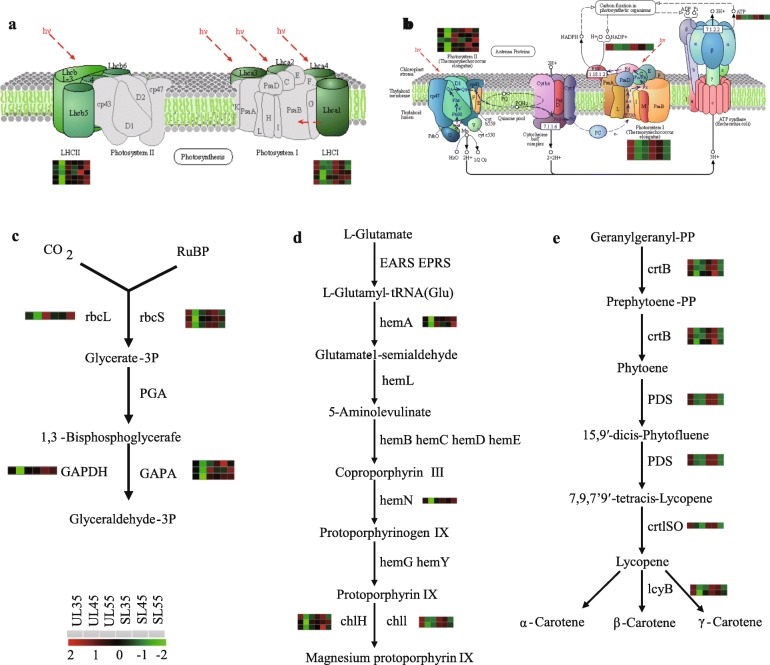
Photosynthesis and light and pigment synthesis pathways of cherry leaves under sheltered covering. **a** DEGs predicted to be involved in the photosynthesis-annotated proteins (ko00196 photosynthesis-antenna protiens) [24], **b** photosynthesis (ko00195 photosynthesis) [24], **c** carbon fixation in photosynthetic organisms, **d** prophyrin and chlorophyl metabolism, and **e** carotenoid biosynthesis. Responsive progression of gene expression under sheltered covering C compared with the unsheltered conditions, indicated in six-box strings (including UL35, 45, 55, LR35, 45, and 55). Heat maps were drawn using log2-transformed FPKM values

To clarify the molecular adaptability of cherry trees to the microclimatic change during fruit development upon exposure to the shelter covering, genes involved in environmental sensitivity were screened out from the filtered DEGs for further investigation (Figs. 7 and 8). Most of the DEGs encoding antenna proteins, or proteins involved in electron transport, reaction center in photosystem I (PSI) and photosystem II (PSII), and components of CO_2_ fixation, were highly expressed in SL compared with UL at DAF45 (Fig. 7a).

More than 70 genes were annotated to three metabolic pathways: photosynthesis-antenna proteins (ko00196), photosynthesis (ko00195), and carbon fixation in photosynthetic organisms (ko00710). Therefore, we focused on the transcriptional levels of those genes closely related to the photosynthetic efficiency (Fig. 7a). All annotated DEGs of light-harvesting Chl a/b binding protein complex I and II (*LHCs*), namely, 17 genes encoding the Chl A/B binding protein complex I (*Lcha1*, *Lcha2*, *Lcha3, Lcha4*, and *Lcha5*) and 20 genes encoding Chl A/B binding protein complex II (*Lchb1*, *Lchb2*, *Lchb3*, *Lchb4*, *Lchb5*, *Lchb6*, and *Lchb7*), were up-regulated in SL at DAF45 (Figs. 7a, 8a). Moreover, 13 genes encoding proteins involved in reaction center and electron transport in photosynthesis, including the PSI reaction center subunit X (*PsaK*), reaction center subunit VI (*PsaH*), PSI reaction center subunit *PsaN* (*PsaN*), PSII oxygen-evolving enhancer protein 2 (*PsbP*), PSII 10 kDa protein (*PsbR*), PSII repair protein *Psb27-H1* (*Psb27*), ferredoxin of photosynthetic electron transport (*PetF*), and H^+^/Na^+^-transporting *ATPase* subunit beta (*AtpF*), were up-regulated under sheltered covering (Figs. 7a, 8b), whereas one gene of the cytochrome f complex (*PetA*) and one gene of H^+^/Na^+^-transporting *ATPase* subunit alpha (*ATPF1AI*) had lower transcription levels. In the carbon fixation pathway, D-ribulose 1,5-bisphosphate (RuBP) and CO_2_ produced 3-phosphate-glycerate (3-PGA) under the action of ribulose-bisphosphate carboxylase large chain (*rcbL*) and ribulose-bisphosphate carboxylase small chain (*rcbS*); then, 3-PGA was reduced to glyceraldehyde-3P (3-PGAld) by glyceraldehyde 3-phosphate dehydrogenase (*GADPH*) and glyceraldehyde-3-phosphate dehydrogenase (*GAPA*), which completed the energy storage process of photosynthesis and increased the production and accumulation of photosynthate. Among them, one gene of *rbcL*, nine genes of *rbcS*, one gene of *GADPH*, and seven genes of *GAPA* were significantly up-regulated under sheltered covering. Notably, the expression of *rbcL* and *GAPA* was up-regulated ≥10-fold (Fig. 7a, 8c).

**Fig. 9:**
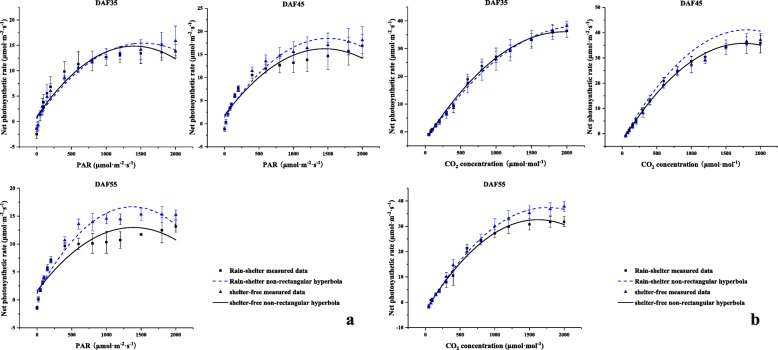
**a** PAR-Pn response curve of the leaves subjected to rain-shelter covering. **b** CO_2_-Pn response curve of the leaves subjected to sheltered covering

In combination with the PAR-Pn and CO_2_-Pn curves, the Pn of sheltered leaves was lower, but there were no significant differences between the first two stages of fruit development (Fig. 9). The AQY and ACE of sheltered leaves visibly increased by 13.0 and 23.5%, respectively; meanwhile, the LCP and CCP parameters decreased to 13.87 and 75.62 μmol·m^− 2^·s^− 1^ at DAF45 (Additional file 8: Table S7). In general, combined with photosynthetic characteristics and transcriptomic results of leaves, the findings illustrated that sheltered leaves had stronger abilities to capture and utilize the weak light, while maintaining stable CO_2_ utilization efficiency. This indicated the possibility of good adaptation to the weak light conditions under the sheltered covering in a short time.

### Expression of genes encoding photosynthetic pigments in sheltered leaves

**Fig. 10:**
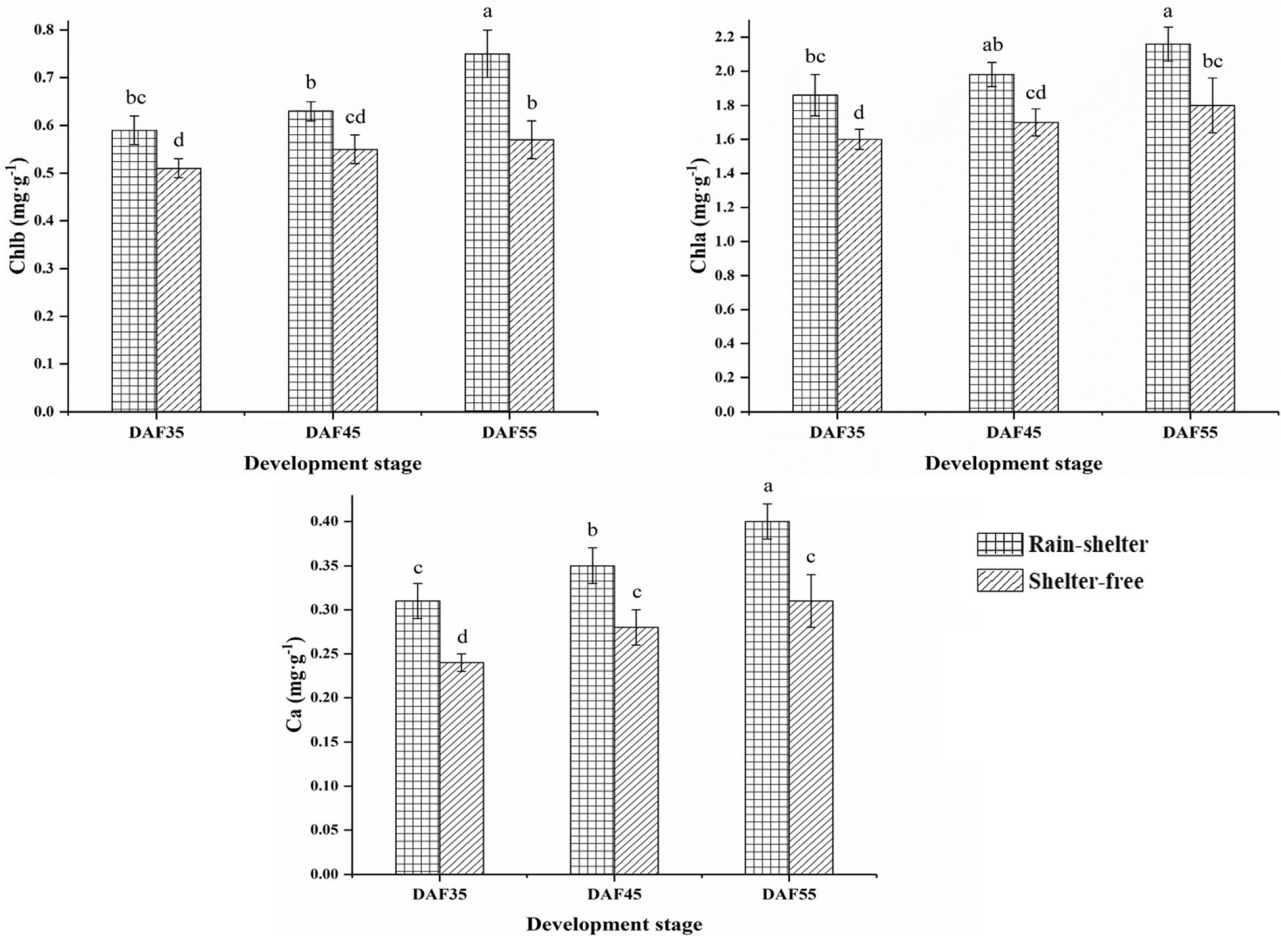
The contents of Chl a, Chl b, and carotenoid of leaves under sheltered covering. The vertical bars represent means of three trees (30 leaves·tree^− 1^). The values marked with different letters are significantly different at *P* < 0.05

The adaptability of plants to weak light is inextricably related to the photosynthetic pigmentation synthesis pathway [25]. In this study, 34 genes involved in porphyrin and chlorophyll metabolism (ko00860) and one gene related to each of terpenoid backbone biosynthesis (ko00900) and carotenoid biosynthesis (ko00906) in sheltered leaves were up-regulated at DAF45 (Fig. 7b). Regarding the DEGs annotated to Chl synthesis and carotenoid biosynthesis, one gene encoding geranylgeranyl diphosphate reductase (*CHLP*), one gene encoding oxygen-independent coproporphyrinogen III oxidase (*hemN*), two genes encoding glutamyl-tRNA reductase (*hemA*), 23 genes encoding magnesium chelatase subunit H (*ChlH*), and two genes encoding magnesium chelatase subunit I (*ChlI*), among others, were up-regulated under sheltered covering at DAF45 (Fig. 8d). Additionally, four genes of 15-cis-phytoene synthase (*crtB*), two genes of 15-cis-phytoene desaturase (*PDS*), one gene of prolycopene isomerase (*crtISO*), and two genes of lycopene beta-cyclase (*lcyB*) were up-regulated under sheltered covering at DAF45 (Fig. 8e). The contents of photosynthetic pigments Chl a, Chl b and Car tended to increase under sheltered covering. Moreover, the contents were higher than those under Cont; for example, Chl a increased by 14 to 16.7%, Chl b by 13.6 to 24%, and Car consistently increased by 20 to 22.6% under sheltered covering (Fig. 10). The transcription levels of genes related to Chl and carotenoid synthesis in the sheltered leaves also fully confirmed that they would not be adversely affected by the sheltered covering (Fig. 7b, 8); conversely, sheltered covering would enhance the gene transcription levels, thereby increasing the pigment content.

### Expression of genes involved in antioxidant systems in sheltered leaves

ROS scavenging enzymes such as *SOD*, catalase (*CAT*), peroxidase (*POD*), and ascorbate peroxidase (*APX*), as well as nonenzymatic antioxidants (glutathione, carotenoids, etc.) are essential for ROS detoxification [26]. Malondialdehyde (MDA) can be used as an indicator of lipid peroxidation under different stress conditions. Transcription levels of multiple genes relating to antioxidant capacity including peroxisome (ko04146), phenylpropanoid biosynthesis (ko00940), glutathione metabolism (ko00053), and carotenoid biosynthesis (ko00906), fluctuated slightly with the microclimatic conditions (Fig. 7c). The expression of most antioxidant-related genes was clearly higher in SL than in UL at DAF45; for example, eight genes involved in peroxisome (encoding *SOD*, and *CAT*), 32 genes involved in phenylpropanoid biosynthesis [encoding trans-cinnamate 4-monooxygenase, *CYP*; phenylalanine ammonia-lyase, *PAL*; 4-coumarate-CoA ligase, *4CL*; cinnamyl-alcohol dehydrogenase, *CAD*; caffeic acid 3-O-methyltransferase, *COMT*; 5-O-(4-coumaroyl)-D-quinate-3′-monooxygenase, *C3’H*; caffeoyl-CoA O-methyltransferase, *CCoAOMT*; and *POD*]; eight genes involved in glutathione metabolism (encoding glutathione synthetase, *GSS*; glutathione reductase, *GSR*; and glutathione S-transferase, *GST*); and 16 genes involved in carotenoid biosynthesis (encoding zeaxanthin epoxidase, *ZEP*; violaxanthin de-epoxidase, *VED*; beta-carotene 3-hydroxylase, *CrtZ*; beta-ring hydroxylase, *LUT5*; lycopene beta-cyclase, and *lcyB*; and abscisic acid 8′-hydroxylase, *CYP707A*) showed markedly higher expression in SL than in UL at DAF45 (Fig. 7c).

**Fig. 11:**
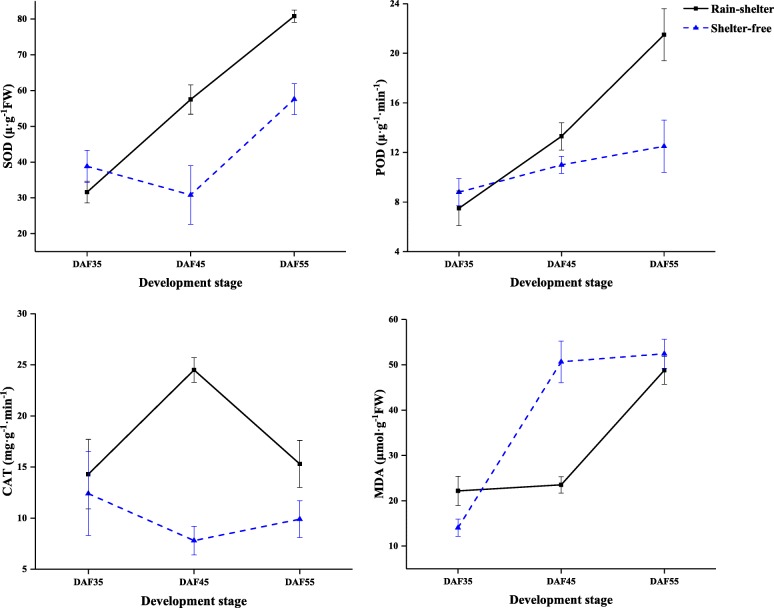
Effect of rain-shelter conditions on antioxidant enzyme activities and MDA contents in cherry leaves. The values represent mean ± SD of three trees (30 leaves·tree^− 1^)

The antioxidant enzyme activities and MDA content in leaves also changed with the change of microclimate associated with sheltered covering. At DAF35, there was no remarkable difference in the activities of *SOD*, *POD*, and *CAT* of SL and UL; however, the MDA content was higher under sheltered covering. Moreover, the activities of antioxidant enzymes were higher than under shelter-free conditions at DAF45, whereas the MDA content was lower than in UL, with the *POD*, *SOD*, and *CAT* activities increasing by 1.7-, 1.9-, and 1.3-fold, respectively (Fig. 11). These results showed that the activities of antioxidant enzymes in leaves increased during RSC, which maintained the ability of plants to scavenge ROS, and prevented the damage of membrane lipid peroxidation to leaves, thus preserving photosynthetic efficiency. The results showed the same trend as the results of gene expression levels.

### Expression of genes associated with anthocyanin synthesis in sheltered fruits

**Fig. 12:**
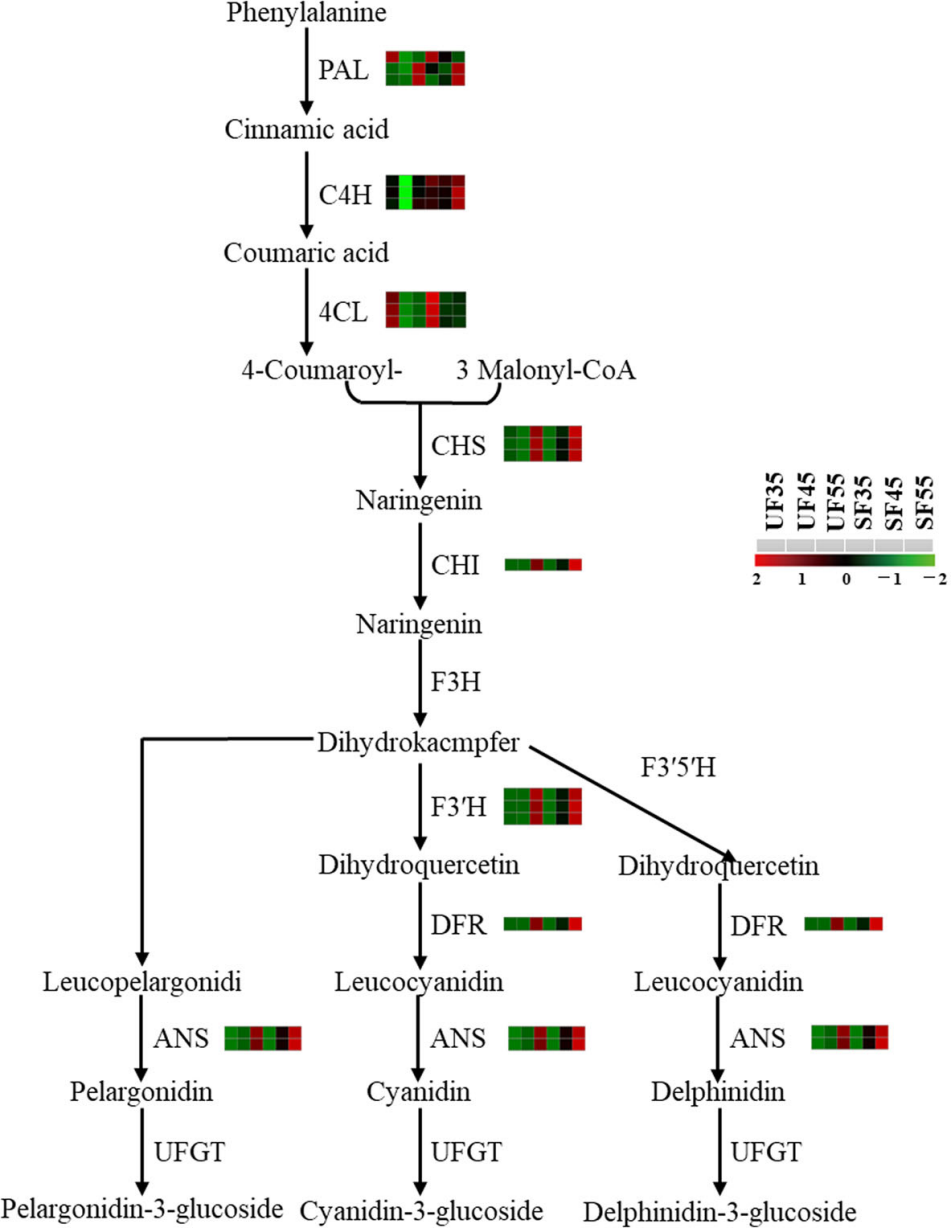
Biosynthetic pathway of anthocyanidins of cherry fruits under rain-shelter covering. Responsive progression of gene expression under sheltered conditions compared with that under unsheltered conditions, indicated in six-box strings (including UF35, 45, 55, SF35, 45, and 55). Heat maps were drawn using log2-transformed FPKM values

The anthocyanin content of mature cherry fruits under sheltered covering increased significantly compared with that of mature cherry fruits under unsheltered covering [22]. The up-regulated genes of fruits were mainly involved in phenylpropanoid biosynthesis (ko00940) and flavonoid biosynthesis (ko00941). The transcriptional levels of more than 25 genes in sheltered fruits began to increase from DAF35 until fruit ripening (Fig. 7d); these included 12 genes involved in phenylpropanoid biosynthesis, with one gene of *PAL*, six genes of *C4H*, and five genes of *4CL*. Among these, the expressions of *C4H* and *4CL* were up-regulated more than 8.5- and 6.1-fold, respectively. Moreover, for 13 genes involved in flavonoid biosynthesis encoding chalcone synthase (*CHS*), chalcone isomerase (*CHI*), flavanone 3′-hydroxylase (*F3’H*), dihydro-flavonol 4-reductase (*DFR*), and anthocyanidin synthase (*ANS*), the expressions were remarkably higher in the sheltered fruits at DAF45 (Fig. 12). The results showed that the synthesis of anthocyanin could be accelerated and the accumulation cycle could be prolonged in the sheltered fruits.

## Discussion

### Regulation highlighted photosynthetic adaptation under shelter covering

The alteration of the physiological characteristics of leaves/fruits may be involved in adaptive responses to changes in microclimate, and the transcriptional regulation of genes is the molecular basis for plant adaptation to shifts in environmental factors such as light and temperature. Photosynthesis is pivotal for plant growth, yield [27] as well as fruit quality; therefore, agronomic techniques are generally particularly focused on improving photosynthesis. Under low light intensity, the photosynthetic efficiency of plants mainly depends on the adaptability of light-harvesting apparatus to the microclimatic conditions, in which PSI/PSII represents the adaptability of plants to low light [28]. *LHCs* in PSI and PSII play important roles in light-harvesting and light protection [29]. *Lhcb* (PSII) was reported to be transcriptionally amplified under low light intensity to enhance photon capture [30]. The differentially expressed transcripts of plant *LHCs* in response to low light stress might play collective roles in maintaining the normal metabolic activities under low light intensity; for example, the genes encoding *Lchbs* from the low-light-tolerant rice cultivar were up-regulated under low light intensity [15]; in the current study, all the DEGs associated with the members of *LHCs*, such as *Lcha* and *Lchb*, were up-regulated in cherry leaves under shelter covering. A total of 37 genes were involved in the regulation of light capture protein expression (Fig. 7a). Compared with the unsheltered leaves, nine genes encoding *Lcha2* and nine genes encoding *Lchb1* were up-regulated; their highest expression levels were increased by 6-fold (*Lcha2*) and 11-fold (*Lchb1*). In addition, *Psb27*, which is essential for energy metabolism and effective recovery of photodamaged PSII complex [31], was also up-regulated in the sheltered leaves. *PsbS* might stabilize the PSII–LHCII supercomplex structure and could improve electron transmission efficiency [32]; in the current study, *PsbS* was up-regulated in the sheltered leaves (Fig. 7a). During the light reaction of photosynthesis, genes encoding LCHs were up-regulated, among which, *Lchbs* promoted the absorption and transmission of light energy under low light conditions, and the light capture area and light capture efficiency were also improved. In addition, the photosynthetic characteristics of sheltered leaves showed that LCP decreased and AQY increased (Additional file 8: Table S7). It is speculated that these genes play important roles in regulating adaptation leaf adaption to the low light environment associated with the rain-shelter covering, by increasing the efficiency of light absorption and electronic transmission, leading to the improvement of weak light utility.

Rubisco (*rbcL* and *rbcS*) is a bifunctional enzyme located in the chloroplast stoma that catalyzes photosynthetic CO_2_ fixation to form ribulose-1,5-bisphosphate (RuBP) [33]; the transcriptional levels of *rbcL* and *rbcS* genes could directly reflect the photosynthetic efficiency of leaves [34]. Available evidences have justified that the activation status of *Rubisco* is affected by light intensity [35]; therefore, the up-regulation of the *rbcL* and *rbcS* in cherry leaves under sheltered covering might play a crucial role in CO_2_ fixation (Fig. 7a). During the CO_2_ assimilation in photosynthesis, genes encoding *Rubisco* (*rbcL* and *rbcS*) were found to be up-regulated; among them, the gene expression of *rbcL* increased by 10.8-fold; nine genes of *rbcS* were also up-regulated (Fig. 8c). Moreover, in terms of the photosynthetic characteristics of sheltered leaves, it was shown that CCP decreased and ACE increased (Additional file 8: Table S7). These results indicated that the enhancement of CO_2_ assimilation under low light intensity could be ascribed as the up-regulation of the *Rubisco*-related genes, which play a significant role in the leaf adaptation to the sheltered covering and provide a material basis for vegetative growth and reproductive growth of plants.

Previous studies indicated that the mechanism of Chl synthesis plays a crucial role in adaptation to low-light conditions [11]. Chl synthesis directly affects photosynthetic efficiency [36], and Chl b is a prerequisite for the stable existence of light-harvesting complex protein (LHCP) [37]. The increase of Chl b was found to be beneficial for increasing LHCP and the absorption of short-wavelength light, thus effectively improved the illumination ability of plants under low light conditions [37]. Furthermore, an obvious increase of Chl b in the low-light-tolerant rice cultivar was beneficial to capture light energy and drive electron transfer under low light, thereby, maintain a high photosynthetic rate [16, 38]. In current case, the the contents (mg·g^− 1^) of Chl b in sheltered leaves were also significantly increased in comparison to those of the unsheltered (Fig. 9), which was beneficial to the stability of leaf LHCP, thus to enhance the utilization of light energy under weak light. The *HemA*, *HemN*, *ChlH* and *ChlL* genes play key roles in plant chlorophyll synthesis. Among them, ChlH is a multifunctional protein with roles in plastid-to-nucleus and plant hormone signal transduction pathways. ChlH accelerated the catalytic activity of ChlM, which catalyzes the conversion of Mg-Proto to Mg-protoporphyrin IX monomethyl ester; this is the key enzyme for chlorophyll synthesis, finally resulting in the synthesis of chlorophyll [39]. In the chlorophyll synthesis pathway of sheltered leaves in current research, the expressions of genes encoding *HemA*, *HemN*, *ChlH*, and *ChlL* were up-regulated in the sheltered leaves. In addition, 23 genes encoding *ChlH* were up-regulated (Fig. 7b). Therefore, the expression of multiple genes related to Chl synthesis were enhanced under the shelter covering, and the increase of Chl b content improved the LHCP status, leading to the elevation in photosynthetic efficiency of the sheltered leaves (Fig. 10).

### Regulation of antioxidant capacity under shelter covering

ROS are produced by plants under stress, which can lead to photoinhibition and photooxidative damage of PSII and PSI [40]. ROS accumulation was shown to be regulated by a series of antioxidant compound; the expression of genes involved in the antioxidant system in plants was found to be diversely inhibited under light stress [41]. The antioxidant enzymes (*SOD*, *CAT*, *POD*, etc.) and nonenzymatic antioxidants (lutein, lignin, glutathione, anthocyanins, etc.) play various roles in the response to abiotic stresses [41]. Under low light conditions, low-light-tolerant rice cultivars were shown to maintain their carbohydrate production level by holding an effective photosynthetic rate and oxygen resistance [15]. Similarly, the activities of antioxidant enzymes in sheltered leaves were higher, which could avoid membrane lipid peroxidation and help maintain photosynthetic efficiency (Fig. 11). Meanwhile, one gene for *SOD*, two genes for *CAT*, and six genes for *POD* were found to be up-regulated, whereas five genes were inhibited in the leaves from the sheltered covering (Fig. 7c). Therefore, the improvement of photosynthetic capacity of sheltered leaves might be considerably contributed to ROS scavenging.

Carotenoids may capture light energy, also, it play a key role in light protection; β-carotene participates in the energy-transfer process and plays an essential role in scavenging singlet oxygen in the photosynthetic reaction centers [42]. In the carotenoid synthesis pathway, nine genes encoding *crtB*, *PDS*, *crtISO*, and *lcyB* were up-regulated in rain-sheltered leaves (Fig. 8d), and the level of carotene was higher in the sheltered leaves (Fig. 10). Furthermore, lutein has the photoprotective function of quenching triplet Chl and excited singlet Chl in light-harvesting antenna systems [43], which provides antioxidant activity by scavenging oxides, thus preventing lipid peroxidation [44]. In the current study, five genes for *LUT5*, *crtZ*, and *lcyB* that synthesize lutein were up-regulated in sheltered leaves. These results further proved that the sheltered leaves enhanced the genes expression of carotenoid synthesis, and somehow promoted photosynthesis.

It is reported that the genes encoding *PAL*, *4CL, CYP* and *CCoAOMT* in phenylpropanoid pathway in shade tolerant soybean cultivar were up-regulated in low light environment [45]. Similarly, 14 genes for *PAL*, *4CL*, and *CYP* were found to be up-regulated under the sheltered leaves herein (Fig. 7c). Glutathione (GSH) can be used as an electron donor to inactivate free radicals, or as a cofactor of several antioxidant enzymes [46] for the detoxification of hydrogen peroxide produced under different stress conditions [47]; in this study, genes for *GSS* and *GSR* were also up-regulated in cherry leaves under the sheltered covering (Fig. 7c).

Based on the above-mentioned results, the antioxidant capacity of sheltered leaves was integrated with the antioxidant enzymes and a variety of nonenzymatic antioxidants to maximize the protection effect, which reduce the negative effect of low light intensity, somewhat maintaining higher photosynthetic efficiency of the sheltered leaves.

### Regulation of anthocyanin biosynthesis under shelter covering

Solar radiation, air humidity, and air temperature in the sheltered covering were reported to be affected by a rain-shelter covering [48]. Anthocyanin accumulation was also influenced by microenvironmental changes [49]. Meanwhile, the light quality could affect the synthesis of anthocyanin by regulating the expression patterns of related genes [50]. Therefore, anthocyanin content is governed by the differential expression of corresponding genes in a metabolic network, which was ascribed to the change in environmental factors [51]. Available evidence shows that *VvPAL* plays a key role in the phenylpropane biosynthesis pathway (pathway upstream of anthocyanin synthesis) by catalyzing phenylalanine to cinnamic acid [19]. Structural genes involved in anthocyanin biosynthesis in *Arabidopsis thaliana* shown to be enzymes expressed in the initial and late stages of the regulation of the flavonoid biosynthesis pathway (pathway downstream of anthocyanin synthesis), such as *CHS* and *CHI* (initial stage), and *DFR* and *ANS* (late stage) are essential for anthocyanin biosynthesis [52]. In addition, *CHS* catalyzes the initial steps of the phenylpropanoid pathway branching to the flavonoid pathway, which is particularly important for the synthesis and accumulation of fruit anthocyanin [53]. In sweet cherry, DEGs including *PAL*, *4CL*, *CHS*, *CHI*, *F3’H*, *DFR*, and *ANS* were confirmed to be involved in anthocyanin synthesis [54]. With the progression of cherry fruit ripening, the genes involved in anthocyanin synthesis were presumably up-regulated under unsheltered conditions [20]. In another study, the levels of phenolic compounds in grape peel under shelter covering were found to be decreased in comparison with those under shelter-free conditions [55]. In addition, a previous study, it was proven that, in grapes under the sheltered covering, the transcript abundance of *VvF3’5’H* and *VvF3’H* was significantly reduced [56], which contrasts with the results of the current study. In this study, these DEGs involved in phenylpropanoid and flavonoid biosynthesis, such as *PAL*, *C4H*, *4CL*, *CHS*, and *CHI*, were up-regulated under covered conditions (Fig. 7d). Among these, the expression of three genes encoding *4CL* increased over 3.5-fold and that of one gene encoding *C4H* increased 8.5-fold. In the flavonoid biosynthesis pathway, compared with the case for fruit grown under shelter-free conditions (DAF45), DEGs encoding *CHS*, *CHI*, *F3’H*, *DFR*, and *ANS* were up-regulated, of which the expression levels of five genes encoding *CHS* increased more than 3.5-fold (Fig. 12). Strong evidence showed that, compared with the unsheltered sweet cherry, the average level of phenols in covered sweet cherry increased by 14%. The results demonstrated that phenolics present under covered conditions can be explained by the larger temperature fluctuations and moderate but not excessive heat stress under the lower-light conditions under the cover [9]. Previous research also showed that anthocyanin synthesis was restricted by upstream metabolites (such as sugar) during fruit development, which proved that anthocyanin accumulation is closely related to photo-assimilates [57]. Furthermore, the results for grapes proved that the increase in anthocyanin content was attributable to a prolonged life of functional leaves, an increase in assimilative products of leaves, and an increase in the soluble solid content of fruits under sheltered covering; ultimately, the content of anthocyanin was also increased [21]. From the results of transcriptomic regulation in the current study, anthocyanin synthesis of fruit under sheltered covering increased the anthocyanin content of fruit by synergizing with key enzymes in upstream/downstream pathways. The synthesis and accumulation of anthocyanins could be increased by increasing the upregulation of genes involved in phenylpropane biosynthesis and flavonoid biosynthesis. Of course, this process was also inseparable from the adaptation of leaves to weak light, which provided assimilation products for the synthesis of fruit anthocyanins.

Collectively, cherry trees could improve photosynthesis and antioxidant capacity by regulating the genes involved in light capture (*LCHs*), carbon dioxide fixation (*Rubisco*), photosynthetic pigment synthesis (*ChlH*), and the antioxidant system (carotenoids and phenolics), and ultimately enhance their adaptability to low light intensity under sheltered covering.

## Conclusion

In summary, we produced some new transcriptome information from Chinese cherry with shelter covering using PacBio and Illumina sequencing in cherry leaves and fruits at three developmental stages under control and rain-shelter covering. The adaptive regulation of cherry leaves and fruits under the sheltered covering was mainly associated with their physiological characteristics (such as LCP, CCP, and Chl content) and transcriptome expression profiles. Also, the transcriptome data confirmed that the adaptation mechanism of leaves to low light was mainly achieved by regulating the gene transcription profiles of LHCP synthesis, photosynthetic electron transfer, CO_2_ fixation, as well as photosynthetic pigment and antioxidant synthesis. Moreover, the sheltered leaves maintained a higher ability for light capture under low light intensity by actively up-regulating *Lcha* and *Lchb* expression, promoted CO_2_ fixation by elevating the expressions of *rbcL* and *rbcS*, and enhanced the expression of *chlH*, thereby improved Chl synthesis. Additionally, improvement in the antioxidants biosynthesis of carotenoid, phenylpropanoid, and glutathione could comprehensively ensure leaves produce enough carbohydrates under low light intensity. Finally, the genes related to anthocyanin biosynthesis, e.g. *PAL*, *C4H*, *4CL*, *CHS*, *CHI*, *F3’H*, *DFR*, and *ANS* were significantly up-regulated in the fruits under the sheltered covering, which might contributed to the anthocyanin accumulation in sheltered fruits. The present study and its findings can serve as valuable resources for future genomic studies on Chinese cherries. The DEG data may also provide useful candidate genes to elucidate the adaptation mechanism of Chinese cherries and other varieties to low-light condition and other abiotic stress.

## Methods

### Plant materials

The trials were conducted in 2016–2018 in an orchard located at Fuquan, Guizhou Province, P.R. China (latitude 26°70′ N, longitude 107°51′ E), which is characterized by a subtropical monsoon climate. At the site, the average temperature is 14 °C, the relative humidity is 88%, and the annual total precipitation is 1220 mm. Five-year-old Chinese cherry trees (“Manaohong” cultivar) were spaced at 3 m between each row and each tree, with the open-center model of trunks and branches. All the trees were subjected to management using the same integrated technique. The trials were conducted with by covering with colorless polyethylene film (semi-covered to ensure ventilation), with a transmission rate of approximately 70%, which was applied from before blooming until fruit harvest. The length, width, and height of the steel frame shelter were 30 m, 10 m, and 4 m (above the ground), respectively. Unsheltered trees were set as a control (Cont). Three trees each under sheltered and unsheltered conditions were labeled for sample collection on the 35th, 45th, and 55th days after flowering (DAF35, DAF45, and DAF55, respectively). Samples of unsheltered leaves (UL35, 45, and 55), unsheltered fruits (UF35, 45, and 55), sheltered leaves (SL35, 45, and 55), and sheltered fruits (SF35, 45, and 55) were collected; immediately frozen in liquid nitrogen; and stored at − 80 °C for physiological indicator measurement and RNA isolation.

### Physiological characteristics of leaves under rain-shelter covering

The photosynthesis light-response (PAR-Pn) and CO_2_-response (CO_2_-Pn) curves under shelter covering and shelter-free conditions were measured between 9:00 a.m. and 11:30 a.m. on a sunny day using a portable infrared gas analyzer (Li-6400XT; Li-Cor, Inc., Lincoln, NE, USA). The photosynthetic PAR-response and CO_2_-response curves were fitted by the Farquhar mathematical model (Prioul and Chartier, 1977). The AQY and CE were obtained by linear regression under a PAR range of 0–200 μmol·m^− 1^·s^− 1^ and CO_2_ concentration range of 0–200 μmol·mol^− 1^; in addition, the LCP and CCP were calculated. Two new southern branches were selected for each tree (three trees per sheltered covering), and the fifth mature leaf was selected from the base of the shoot.

Thirty mature leaves per tree were sampled to quantify the following parameters, and three biological replicates were performed for each of the sheltered and unsheltered conditions. To minimize the positional effect of the canopy, leaves were harvested from four directions: east, south, west, and north. The contents of Chl a, Chl b, and carotenoids (Car) were determined according to the method described by Shi et al. (2013). The amount of malondialdehyde (MDA) and activity of superoxide dismutase (*SOD*, E.C.1.15.1.1) were measured as documented by Wen et al. (2011). The activities of peroxidase (*POD*, E.C. 1.11.1.7) and catalase (*CAT*, E.C. 1.11.1.6) were determined using a spectrophotometer following a previously described method (Sharma et al., 2016). All absorbances of extracts were measured using a microplate spectrophotometer (Thermo Scientific, MA, USA).

### Total RNA extraction, PacBio and Illumina library construction and sequencing

In total, 36 samples [two sheltered coverings (sheltered and unsheltered) × three stages (DAF35, 45, and 55) × two tissues (leaves and fruits) × three biological replicates] were prepared, and the total RNAs were separately obtained using the QIAGEN RNeasy Plus Mini Kit (Cat. No. 74134). The purity and concentration of RNA were determined using a Nanodrop 2000 spectrophotometer and QUBIT Fluorometer (Life Technologies), and the integrity of RNA was assessed using an Agilent 2100 bioanalyzer (Agilent Technologies).

The qualified RNAs of leaves (UL and SL) and fruits (UF and SF) from three stages (DAF35, 45, and 55) were equivalently mixed into two corresponding libraries. cDNA was synthesized using a SMART PCR cDNA Synthesis Kit (Clontech, Cat. No. 639206). PCR was optimized using a KAPA HiFi PCR Kit; different cDNA fractions (0.5–2 kb, 2–3 kb, and > 3 kb) were classified using the BluePippin size selection system (Sage Science, Beverly, MA, USA). The PCR products were amplified and constructed using SMRTbell Template Prep Kit 1.0 (part.100–259-100) following the vendor’s protocol. The library was sequenced on the PacBio Sequel real-time nanopore sequencer using PacBio Sequel V2.1 with five cells (one cell for fruits and four cells for leaves); 0.5–6 kb mixed libraries were sequenced. In total, 36 qualified RNA samples were used for Illumina sequencing. The RNA samples were used for poly(A)^+^ selection using oligo (dT) magnetic beads, while constructing RNA-Seq libraries. The cDNA libraries were sequenced using PacBio Sequel and HiSeq X Ten at Wuhan Nextomics Biosciences Co., Ltd.

### Data processing and gene function annotation

Sequence data of PacBio Sequel were collected, and the high-quality consensus transcript sequences were obtained using smrtlink 5.0. Subsequently, the iterative clustering for error correction (ICE) algorithm was used to remove redundancy and improve the accuracy of the full-length transcripts. Raw data of Illumina HiSeq were processed through inner Perl scripts. Clean data were obtained by removing reads containing adapters, reads containing poly-N regions, and low-quality reads from the raw data. Meanwhile, the Illumina HiSiq reads were used to correct all full-length transcripts of single-molecule real-time (SMRT) Squel by Lordec [58]. The proofread-corrected sequences after removal of the redundant sequences using CD-HIT-EST [59] were used as reference sequences for further analyses. Gene functions were searched against the databases of NR, COG, Swiss-Prot, KEGG, and GO.

### Differential expression, GO, and KEGG enrichment analysis

Clean reads of all samples were counted and normalized into fragments per kilobase of transcript per million fragments mapped reads (FPKM) value using RSEM (v1.1.12) [60]. DEGs between different treatments were determined using the DESeq package [61]. The FDR values were < 0.05 and an absolute value of log2FoldChange ≥1 (or < − 1) were used to identify genes that were significantly differentially expressed. The GO functional classification statistics of DEGs involved the use of the WEGO software [62]. Metabolic pathway assignments of DEGs were implemented based on the KEGG Orthology database (http://www.genome.ad.jp/kegg/) using the KAAS system (http://www.genome.jp/tools/kaas/). The overall pathway analyses were mainly based on the tool Mercator4 and Mapman4 [63]. MapMan BIN was inferred based on both automatic and manual annotations. The different expression patterns of DEGs among the leaves and fruits were assessed using the R language, Cluster, Fpc, Ggplot2, and Reshape2 packages; the clustering method was K-means clustering.

### Quantitative real-time PCR (qRT-PCR) analysis

The expression levels of samples under the different conditions, including leaves and fruits, were validated by qRT-PCR using PowerUp™ SYBR Green Master Mix (ThermoFisher, Chongqing, China) in a volume of 10 μL, which contained 5 μl of SYBR Green Master Mix, 150 ng (leaf)/100 ng (fruit) of cDNA template, and 0.4 μM of each of the forward and reverse primers. The qRT-PCR amplification was performed as follows: 95 °C for 30 s, followed by 40 cycles of 95 °C for 5 s and 60 °C for 30 s. Three cherry actin genes (*UBCE*, *CYP2*, and *ACT2*) were used as reference genes, as previously reported [64, 65]; the primer sequences are listed in Table S2. Relative gene expression was calculated using the 2^−ΔΔ*Ct*^ method [66] with the CFX Connect™ Real-Time PCR Detection System (Bio-Rad Laboratories, CA, USA). All validations were performed in three biological and technical replicates.

### Statistical analysis

All data were assessed for significant differences using Duncan’s and Tukey’s tests using the SPSS 21.0 statistics package (Chicago, IL, USA). All data are presented as the mean and standard deviation (SD) of at least three replicates. The graphs were constructed with Origin 9.0 (Origin Lab, Northampton, MA, USA).

## Supplementary information

**Additional file 1: Figure S1.** Density of full-length non-chimeric reads (FLNCs).

**Additional file 2: Table S1.** Summary of PacBio Sequel real-time sequencing. The four rows of “SL and UL^1-4^” represent the leaf library, and the row “SF and UF” represent the fruit library. ^a^Polymerase, the original read generated by PacBio Sequel; ^b^Subreads, Post-filter polymerase reads; ^c^Sequencing times of insert; ^d^Number of circular consensus sequences; ^e^Number of full-length non-chimeric.

**Additional file 3: Table S2.** Sequences of primers used for qRT-PCR.

**Additional file 4: Table S3.** Classification of GO annotations (the top 50 GO terms).

**Additional file 5: Table S4.** Classification of KEGG annotation.

**Additional file 6: Table S5.** Statistics of RNA-Seq data.

**Additional file 7: Table S6.** The biochemical pathway of MapMan annotation in leaves and fruits of the rain-sheltered condition.

**Additional file 8: Table S7.** Responses of photosynthesis to different conditions in leaves during different stages. The values represent the mean ± SD of three trees (six leaves·tree^− 1^). The means followed by different letters in the same columns are significantly different at *P* < 0.05.

## Data Availability

The datasets supporting the conclusions of this article are included within the article and its additional files. The datasets used and/or analyzed during the current study are available from the authors on reasonable request (Tian Tian, tiantiangzu@163.com; Guang Qiao, 13518504594@163.com).

## Abbreviations

ANS: Anthocyanidin synthase
BP: Biological process
CC: Cellular component
CCS: Circular consistent sequence
DEG: Differentially expressed genes
FLNC: Full-length non-chimera
GO: Gene Ontology
ICE: Iterative clustering for error
KEGG: Kyoto Encyclopedia of Genes and Genomics
LHCP: Light-collecting complex protein
PAR: Photosynthetically active radiation
PDS: Phytoene desaturase
SD: Standard deviation
SMRT: Single-molecule real-time
