# Supplementary figures and images for: Comparative transcriptome analysis reveals the molecular regulation underlying the adaptive mechanism of cherry (Cerasus pseudocerasus Lindl.) to shelter covering
Source: BMC Plant Biol. 2020 Jan 17;20:27. doi: 10.1186/s12870-019-2224-x (PMC6967096; doi:10.1186/s12870-019-2224-x)

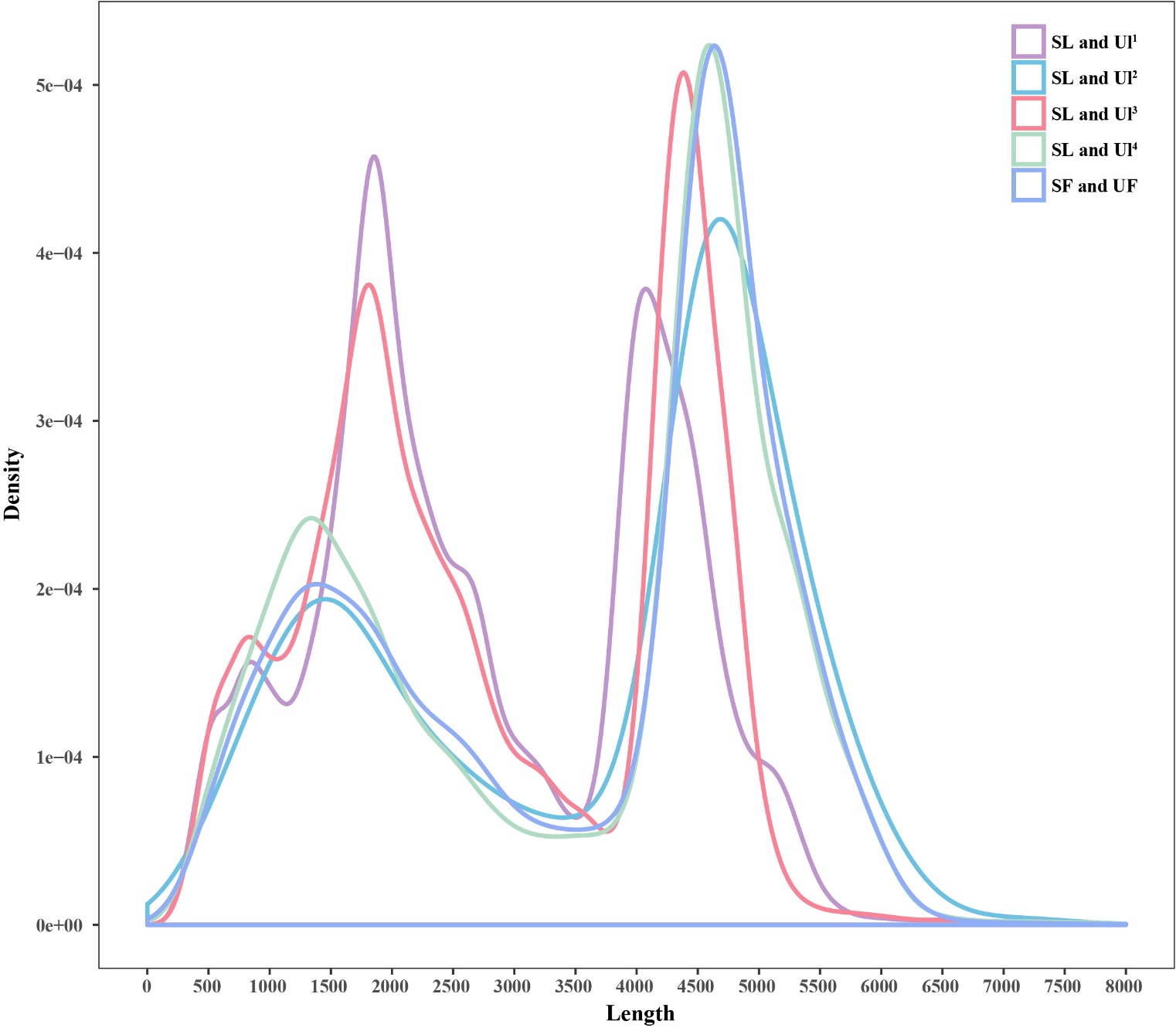


Figure S1

Supplement: Supplementary file 1 — Additional file 1: Figure S1. Density of full-length non-chimeric reads (FLNCs). [file 12870_2019_2224_MOESM1_ESM.docx]
