# Supplementary material for: Comparative transcriptome analysis reveals the molecular regulation underlying the adaptive mechanism of cherry (Cerasus pseudocerasus Lindl.) to shelter covering
Source: BMC Plant Biol. 2020 Jan 17;20:27. doi: 10.1186/s12870-019-2224-x (PMC6967096; doi:10.1186/s12870-019-2224-x)
Supplement: Supplementary file 2 — Additional file 2: Table S1. Summary of PacBio Sequel real-time sequencing. The four rows of “SL and UL1-4” represent the leaf library, and the row “SF and UF” represent the fruit library. aPolymerase, the original read generated by PacBio Sequel; bSubreads, Post-filter polymerase reads; cSequencing times of insert; dNumber of circular consensus sequences; eNumber of full-length non-chimeric. [file 12870_2019_2224_MOESM2_ESM.docx]

Table S1 Summary of PacBio Sequel real-time sequencing

| **Library** | **Polymerase N50 length^a^** | **Subreads N50**  **length^b^** | **Mean number**  **of passes^c^** | **Number of CCS^d^** | **Number of FLNC^e^** | **Mean length**  **of FLNC** |
| --- | --- | --- | --- | --- | --- | --- |
|  |  |  |  |  |  |  |
| SL and UL^1^ | 41,750 | 4,031 | 10.11 | 69,562 | 52,291 | 3,468 |
| SL and UL^2^ | 43,250 | 4,009 | 11.26 | 21,950 | 17,739 | 3,549 |
| SL and UL^3^ | 36,750 | 2,808 | 10.73 | 278,447 | 231,786 | 2,886 |
| SL and UL^4^ | 45,750 | 4,117 | 12.06 | 1,692 | 1,355 | 3,631 |
| SF and UF | 39,750 | 2,742 | 11.49 | 313,688 | 239,624 | 2,834 |
