# Supplementary material for: Comparative transcriptome analysis reveals the molecular regulation underlying the adaptive mechanism of cherry (Cerasus pseudocerasus Lindl.) to shelter covering
Source: BMC Plant Biol. 2020 Jan 17;20:27. doi: 10.1186/s12870-019-2224-x (PMC6967096; doi:10.1186/s12870-019-2224-x)
Supplement: Supplementary file 3 — Additional file 3: Table S2. Sequences of primers used for qRT-PCR. [file 12870_2019_2224_MOESM3_ESM.docx]

Table S2 Sequences of primers used for qRT-PCR.

| Unigene ID | Primer sequence (5'->3') | Functional annotation |
| --- | --- | --- |
| Ye et al., 2014 | forward: CTTCAGAAAGACCCACCCGCATC | ubiquitin-conjugating enzyme, UBCE |
|  | reverse: TACAAGAAAAACACCTCCAGCAAATGG |  |
| Ye et al., 2014 | forward: ACTCCAAAGCGTGTTAGAAAAGG | cyclophilin, CYP2 |
|  | reverse: GTCTCTTCCACCATAACGATAGG |  |
| Zhu et al., 2014 | forward: CCGAATTACGTGGTGGTGGA | Actin 7 (ACT7)/actin 2, ACT2 |
|  | reverse: CCCCAACCGATCAAACCTGA |  |
| >PB.36491.1 | forward: GCCCATAGAAAGGAGGAGACAAA | lycopene beta-cyclase, lcyB |
|  | reverse: AGATGACAAGAATCCGTGCCAAT |  |
| >PB.18981.1 | forward: TATCTTCTTTGGGGCTTATCCG | 15-cis-phytoene desaturase, PDS |
|  | reverse: CTCTCCTGGTTTGTTTGGCATT |  |
| >PB.27515.1 | forward: GCTTGTGTCTCTGCTGCTAACTT | 15-cis-phytoene desaturase, PDS2 |
|  | reverse: GATAATCAACGCAAACCACCTTC |  |
| >PB.32404.6 | forward: GCTATTATGTTGCTGGGACTGTT | 15-cis-phytoene synthase,crtB1 |
|  | reverse: TCCTCCACTTGTCTGTGACCTTT |  |
| >PB.31246.5 | forward: GATGCCAGGAGAGGAAGAGTGTA | 15-cis-phytoene synthase, crtB2 |
|  | reverse: ACTTCTTTGCTTTCCCGACATAA |  |
| >PB.43065.1 | forward: TTGATCCTCTGGGACTTGCTG | light-harvesting complex I chlorophyll a/b binding protein 4, Lhca4 |
|  | reverse: TTCTTGATGTCTTGCCACCGT |  |
| >PB.42224.3 | forward: ACCTCTTGGACCACCTTGACA | light-harvesting complex II chlorophyll a/b binding protein 3, Lhcb1A |
|  | reverse: AAGCCGACACGCAATGAAA |  |
| >PB.27858.1 | forward: CTGGTGACTACGGATGGGACA | light-harvesting complex II chlorophyll a/b binding protein 1, Lhcb1C |
|  | reverse: CTCTGGGCATGGACAAGGTTT |  |
| >PB.26528.3 | forward: TTGGCTGAAGACAATGATGACA | ribulose-bisphosphate carboxylase small chain, rbcS |
|  | reverse: TGCGAGTTACCCCACAAAGAG |  |
| >PB.2780.1 | forward: TGGAATTTGGCACCCTTTAGC | magnesium chelatase subunit H, chlH |
|  | reverse: TGGTATCACCTTAGCCCCTCTT |  |
| >PB.46514.2 | forward: CAGCAGCCACTGATGACCAC | photosystem I subunit X, PsaK |
|  | reverse: CCCAAACCTCCCAGCAAATA |  |
| >PB.45091.1 | forward: CAGCCAGTGCGTTCACATCA | photosystem I subunit PsaN, PsaN |
|  | reverse: GTCCTTGCCTTCGCATTCC |  |
