# Supplementary material for: Comparative transcriptome analysis reveals the molecular regulation underlying the adaptive mechanism of cherry (Cerasus pseudocerasus Lindl.) to shelter covering
Source: BMC Plant Biol. 2020 Jan 17;20:27. doi: 10.1186/s12870-019-2224-x (PMC6967096; doi:10.1186/s12870-019-2224-x)
Supplement: Supplementary file 4 — Additional file 4: Table S3. Classification of GO annotations (the top 50 GO terms). [file 12870_2019_2224_MOESM4_ESM.docx]

Table S3 Classification of GO annotations (the top 50 GO terms)

| **GO ID** | **GO Term** | **Namespace** | **Gene numbers** | |
| --- | --- | --- | --- | --- |
| GO:0005515 | protein binding | molecular_function | | 4812 |
| GO:0005524 | ATP binding | molecular_function | | 3907 |
| GO:0006468 | protein phosphorylation | biological_process | | 2273 |
| GO:0004672 | protein kinase activity | molecular_function | | 2272 |
| GO:0055114 | oxidation-reduction process | biological_process | | 1833 |
| GO:0043531 | ADP binding | molecular_function | | 1725 |
| GO:0016020 | membrane | cellular_component | | 1656 |
| GO:0016021 | integral component of membrane | cellular_component | | 1571 |
| GO:0003676 | nucleic acid binding | molecular_function | | 1265 |
| GO:0007165 | signal transduction | biological_process | | 1255 |
| GO:0003677 | DNA binding | molecular_function | | 1203 |
| GO:0055085 | transmembrane transport | biological_process | | 1181 |
| GO:0008270 | zinc ion binding | molecular_function | | 1057 |
| GO:0006355 | regulation of transcription, DNA-templated | biological_process | | 945 |
| GO:0008152 | metabolic process | biological_process | | 845 |
| GO:0016491 | oxidoreductase activity | molecular_function | | 788 |
| GO:0003824 | catalytic activity | molecular_function | | 766 |
| GO:0005634 | nucleus | cellular_component | | 748 |
| GO:0005975 | carbohydrate metabolic process | biological_process | | 628 |
| GO:0006508 | proteolysis | biological_process | | 589 |
| GO:0006810 | transport | biological_process | | 585 |
| GO:0016887 | ATPase activity | molecular_function | | 504 |
| GO:0046872 | metal ion binding | molecular_function | | 481 |
| GO:0003723 | RNA binding | molecular_function | | 465 |
| GO:0005622 | intracellular | cellular_component | | 420 |
| GO:0003735 | structural constituent of ribosome | molecular_function | | 419 |
| GO:0006412 | translation | biological_process | | 418 |
| GO:0005840 | ribosome | cellular_component | | 408 |
| GO:0003700 | transcription factor activity | molecular_function | | 407 |
| GO:0020037 | heme binding | molecular_function | | 407 |
| GO:0016787 | hydrolase activity | molecular_function | | 402 |
| GO:0005525 | GTP binding | molecular_function | | 388 |
| GO:0004553 | hydrolase activity | molecular_function | | 381 |
| GO:0005506 | iron ion binding | molecular_function | | 330 |
| GO:0006886 | intracellular protein transport | biological_process | | 314 |
| GO:0042626 | ATPase activity | molecular_function | | 299 |
| GO:0005509 | calcium ion binding | molecular_function | | 297 |
| GO:0016705 | oxidoreductase activity | molecular_function | | 291 |
| GO:0015074 | DNA integration | biological_process | | 280 |
| GO:0009058 | biosynthetic process | biological_process | | 279 |
| GO:0003924 | GTPase activity | molecular_function | | 267 |
| GO:0015979 | photosynthesis | biological_process | | 239 |
| GO:0046983 | protein dimerization activity | molecular_function | | 235 |
| GO:0008168 | methyltransferase activity | molecular_function | | 234 |
| GO:0006629 | lipid metabolic process | biological_process | | 223 |
| GO:0006351 | transcription, DNA-templated | biological_process | | 208 |
| GO:0005737 | cytoplasm | cellular_component | | 200 |
| GO:0016192 | vesicle-mediated transport | biological_process | | 195 |
| GO:0016301 | kinase activity | molecular_function | | 190 |
