# Supplementary material for: Comparative transcriptome analysis reveals the molecular regulation underlying the adaptive mechanism of cherry (Cerasus pseudocerasus Lindl.) to shelter covering
Source: BMC Plant Biol. 2020 Jan 17;20:27. doi: 10.1186/s12870-019-2224-x (PMC6967096; doi:10.1186/s12870-019-2224-x)
Supplement: Supplementary file 5 — Additional file 5: Table S4. Classification of KEGG annotation. [file 12870_2019_2224_MOESM5_ESM.docx]

Table S4 Classification of KEGG annotation

| **KEGG_classification** | **KEGG_sub_classification** | **Gene numbers** | |
| --- | --- | --- | --- |
| Cellular Processes | Cell growth and death | | 587 |
| Cellular Processes | Cell motility | | 140 |
| Cellular Processes | Cellular community | | 197 |
| Cellular Processes | Transport and catabolism | | 1067 |
| Environmental Information Processing | Membrane transport | | 176 |
| Environmental Information Processing | Signal transduction | | 1563 |
| Genetic Information Processing | Folding, sorting, and degradation | | 1216 |
| Genetic Information Processing | Replication and repair | | 491 |
| Genetic Information Processing | Transcription | | 756 |
| Genetic Information Processing | Translation | | 1491 |
| Metabolism | Amino acid metabolism | | 1005 |
| Metabolism | Biosynthesis of other secondary metabolites | | 362 |
| Metabolism | Carbohydrate metabolism | | 1719 |
| Metabolism | Energy metabolism | | 1200 |
| Metabolism | Glycan biosynthesis and metabolism | | 331 |
| Metabolism | Lipid metabolism | | 796 |
| Metabolism | Metabolism of cofactors and vitamins | | 652 |
| Metabolism | Metabolism of other amino acids | | 546 |
| Metabolism | Metabolism of terpenoids and polyketides | | 341 |
| Metabolism | Nucleotide metabolism | | 500 |
| Metabolism | Overview | | 1173 |
| Metabolism | Xenobiotics biodegradation and metabolism | | 178 |
| Organismal Systems | Aging | | 291 |
| Organismal Systems | Circulatory system | | 124 |
| Organismal Systems | Development | | 81 |
| Organismal Systems | Digestive system | | 256 |
| Organismal Systems | Endocrine system | | 700 |
| Organismal Systems | Environmental adaptation | | 613 |
| Organismal Systems | Excretory system | | 152 |
| Organismal Systems | Immune system | | 404 |
| Organismal Systems | Nervous system | | 433 |
| Organismal Systems | Sensory system | | 23 |
