# Supplementary material for: Comparative transcriptome analysis reveals the molecular regulation underlying the adaptive mechanism of cherry (Cerasus pseudocerasus Lindl.) to shelter covering
Source: BMC Plant Biol. 2020 Jan 17;20:27. doi: 10.1186/s12870-019-2224-x (PMC6967096; doi:10.1186/s12870-019-2224-x)
Supplement: Supplementary file 6 — Additional file 6: Table S5. Statistics of RNA-Seq data. [file 12870_2019_2224_MOESM6_ESM.docx]

Table S5 Statistics of RNA-Seq data

| **Sample** | **Read Length** | **Clean reads** | **Clean Bases** | **Q30 Rate (%)** |
| --- | --- | --- | --- | --- |
| UL1a | 150 | 52,673,392 | 7,901,008,800 | 86.44 |
| UL1b | 150 | 54,805,772 | 8,220,865,800 | 88.14 |
| UL1c | 150 | 45,129,194 | 6,769,379,100 | 91.13 |
| UL2a | 150 | 44,489,744 | 6,673,461,600 | 91.45 |
| UL2b | 150 | 49,200,622 | 7,380,093,300 | 91.21 |
| UL2c | 150 | 42,526,888 | 6,379,033,200 | 90.55 |
| UL3a | 150 | 47,780,510 | 7,167,076,500 | 91.2 |
| UL3b | 150 | 53,079,164 | 7,961,874,600 | 91.57 |
| UL3c | 150 | 51,913,082 | 7,786,962,300 | 91.84 |
| SL1a | 150 | 49,286,234 | 7,392,935,100 | 88.75 |
| SL1b | 150 | 31,041,784 | 4,656,267,600 | 90.29 |
| SL1c | 150 | 59,903,496 | 8,985,524,400 | 89.39 |
| SL2C | 150 | 54,813,814 | 8,222,072,100 | 87.88 |
| SL2a | 150 | 55,161,836 | 8,274,275,400 | 88.59 |
| SL2b | 150 | 53,887,168 | 8,083,075,200 | 88.21 |
| SL3a | 150 | 44,217,330 | 6,632,599,500 | 90.49 |
| SL3b | 150 | 56,527,248 | 8,479,087,200 | 89.19 |
| SL3c | 150 | 46,071,792 | 6,910,768,800 | 90.46 |
| UF1a | 150 | 41,415,178 | 6,212,276,700 | 91.55 |
| UF1b | 150 | 52,619,268 | 7,892,890,200 | 90.31 |
| UF1c | 150 | 44,391,590 | 6,658,738,500 | 89.54 |
| UF2a | 150 | 55,954,938 | 8,393,240,700 | 90.1 |
| UF2b | 150 | 42,697,036 | 6,404,555,400 | 89.87 |
| UF2c | 150 | 49,223,124 | 7,383,468,600 | 90.58 |
| UF3a | 150 | 39,856,672 | 5,978,500,800 | 89.51 |
| UF3b | 150 | 39,020,436 | 5,853,065,400 | 89.54 |
| UF3c | 150 | 46,430,132 | 6,964,519,800 | 90.16 |
| SF1a | 150 | 48,057,948 | 7,208,692,200 | 91.43 |
| SF1b | 150 | 47,508,882 | 7,126,332,300 | 91.1 |
| SF1c | 150 | 53,305,256 | 7,995,788,400 | 91.53 |
| SF2a | 150 | 42,371,062 | 6,355,659,300 | 90.37 |
| SF2b | 150 | 44,833,538 | 6,725,030,700 | 91.04 |
| SF2c | 150 | 45,198,020 | 6,779,703,000 | 91.23 |
| SF3a | 150 | 52,184,320 | 7,827,648,000 | 91.47 |
| SF3b | 150 | 43,480,676 | 6,522,101,400 | 91.94 |
| SF3c | 150 | 44,166,014 | 6,624,902,100 | 91.31 |
