# Supplementary material for: Comparative transcriptome analysis reveals the molecular regulation underlying the adaptive mechanism of cherry (Cerasus pseudocerasus Lindl.) to shelter covering
Source: BMC Plant Biol. 2020 Jan 17;20:27. doi: 10.1186/s12870-019-2224-x (PMC6967096; doi:10.1186/s12870-019-2224-x)
Supplement: Supplementary file 8 — Additional file 8: Table S7. Responses of photosynthesis to different conditions in leaves during different stages. The values represent the mean ± SD of three trees (six leaves·tree− 1). The means followed by different letters in the same columns are significantly different at P < 0.05. [file 12870_2019_2224_MOESM8_ESM.docx]

Table S7 Responses of photosynthesis to different conditions in leaves during different stages

| **Treatment** | **Development stage** | **AQY** | **LCP/ (μmol·m^−2^·s^−1^)** | **ACE** | **CCP/ (μmol·m^−2^·s^−1^)** |
| --- | --- | --- | --- | --- | --- |
| Rain-shelter | DAF35 | 0.046 ± 0.005a | 17.29 ± 3.16bc | 0.032 ± 0.000b | 82.25 ± 7.62 |
|  | DAF45 | 0.044 ± 0.002bc | 13.87 ± 0.50c | 0.043 ± 0.002a | 75.64 ± 11.19 |
|  | DAF55 | 0.044 ± 0.002bc | 14.51 ± 3.33c | 0.041 ± 0.001a | 84.01 ± 6.29 |
| Unsheltered | DAF35 | 0.038 ± 0.005c | 22.53 ± 5.93a | 0.030 ± 0.005b | 93.22 ± 11.44 |
|  | DAF45 | 0 .039 ± 0.001c | 17.33 ± 0.74bc | 0.035 ± 0.004b | 79.20 ± 11.86 |
|  | DAF55 | 0.041 ± 0.002bc | 15.91 ± 2.51c | 0.040 ± 0.001a | 87.87 ± 7.06 |

The values represent the mean ± SD of three trees (six leaves·tree^−1^). The means followed by different letters in the same columns are significantly different at *P* < 0.05.
